# Nuisance Flooding and Relative Sea-Level Rise: the Importance of Present-Day Land Motion

**DOI:** 10.1038/s41598-017-11544-y

**Published:** 2017-09-11

**Authors:** Makan A. Karegar, Timothy H. Dixon, Rocco Malservisi, Jürgen Kusche, Simon E. Engelhart

**Affiliations:** 1 School of Geosciences, University of South Florida, Tampa, Florida USA; 2 Institute of Geodesy and Geoinformation, University of Bonn, Bonn, Germany; 3 Department of Geosciences, University of Rhode Island, Kingston, Rhode Island USA

## Abstract

Sea-level rise is beginning to cause increased inundation of many low-lying coastal areas. While most of Earth’s coastal areas are at risk, areas that will be affected first are characterized by several additional factors. These include regional oceanographic and meteorological effects and/or land subsidence that cause relative sea level to rise faster than the global average. For catastrophic coastal flooding, when wind-driven storm surge inundates large areas, the relative contribution of sea-level rise to the frequency of these events is difficult to evaluate. For small scale “nuisance flooding,” often associated with high tides, recent increases in frequency are more clearly linked to sea-level rise and global warming. While both types of flooding are likely to increase in the future, only nuisance flooding is an early indicator of areas that will eventually experience increased catastrophic flooding and land loss. Here we assess the frequency and location of nuisance flooding along the eastern seaboard of North America. We show that vertical land motion induced by recent anthropogenic activity and glacial isostatic adjustment are contributing factors for increased nuisance flooding. Our results have implications for flood susceptibility, forecasting and mitigation, including management of groundwater extraction from coastal aquifers.

## Introduction

While it is not currently possible to predict the coastal locations that will be flooded by major storms and hurricanes in the future, the timing and location of nuisance flooding can be predicted with some accuracy^1–4^. Timing is a strong function of local tides, while location is determined by places where land elevation is currently close to local sea level, and hence can be temporarily flooded when the sea surface exceeds some threshold elevation (termed the nuisance flood level). Even moderate amounts of relative sea-level rise (RSLR; the combination of land motion and absolute sea-level rise) can affect this threshold, and hence the nuisance flood frequency.

We investigate the frequency and location of nuisance flooding along the eastern seaboard of North America, and compare these to various processes affecting relative sea level on different time scales. We use rates of vertical land motion measured by Global Positioning System (GPS), rates of recent (1990 - present) RSLR from tide gauges, groundwater-level change from monitoring wells, water storage from the GRACE satellite gravity mission, the geologic rate of RSLR, and Glacial Isostatic Adjustment (GIA) models. We use these diverse data to assess the various factors that influence nuisance-flooding events. In addition to the well-known influence of GIA (described below) we show that recent anthropogenic activities (e.g., groundwater extraction and surface water storage by dams) and corresponding vertical land motions can also influence whether or not a given area experiences nuisance flooding. Parts of the Atlantic coast of North America may also be experiencing sea-level changes in response to large-scale changes in North Atlantic circulation^5–12^. Since tide gauges measure the combined effect of water-level change and vertical land motion, an accurate description and understanding of relevant land motions is important if we hope to use tide-gauge data to describe and understand recent inter-annual to decadal-scale oceanographic changes^13^.

## Data Sets and Observations

**Figure 1:**
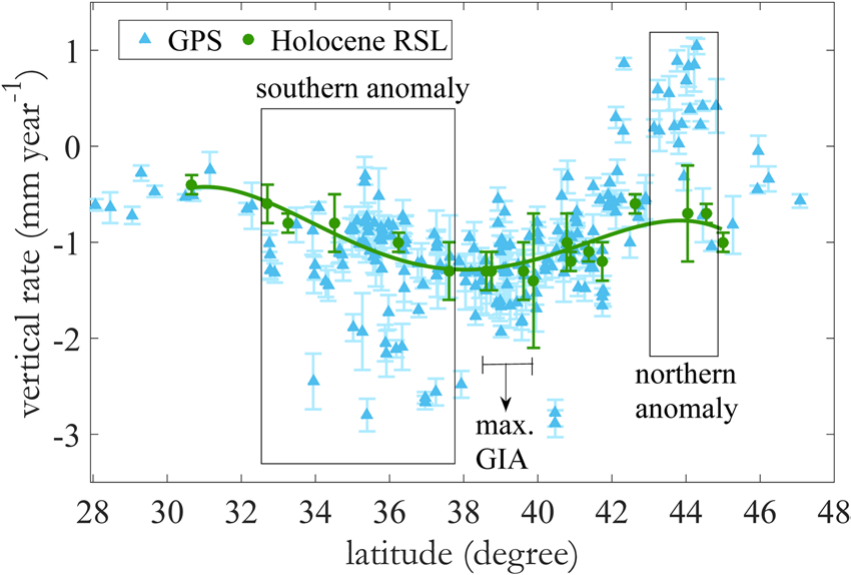
Comparison of vertical land motion from GPS (triangles) and late Holocene RSL data (circles) as a function of latitude. The green solid curve is 4th-order polynomial fit to the geologic rates and shows the general pattern of GIA-induced subsidence along the Atlantic Coast of North America. Error bars are 1σ. The GPS rate uncertainties account for time-correlated noise using the Allan Variance of rates method^64, 65^. Uncertainties in the late Holocene RSL rates are derived from propagation of errors^15^. There is a good agreement (within data uncertainty) between estimates of vertical land motion from GPS and geologic data, except for two regions. Between 37.5°N and 32.5°N (southern anomaly) the GPS data show present-day subsidence rates that are approximately double the long-term geologic rates (see also Fig. S2). In Maine, between 45°N-43°N (northern anomaly), the GPS data show uplift whereas geologic data show subsidence. “max. GIA” indicates the maximum subsidence due to collapse of the peripheral bulge associated with the Laurentide ice sheet.

We use GPS data spanning the last one to two decades to estimate recent vertical land motions over this period^14^. These data can be compared to longer term geological data based on radiocarbon dating of basal peat deposits, which define rates of RSLR averaged over the late Holocene period^15^. These data assume that late Holocene glacier meltwater input is minimal from 4 ka B.P. until 1900 A.D., and hence provide estimates of spatially variable vertical land motion dominated by GIA over this time frame. Melting of the polar ice sheets could affect RSLS rates inferred from the geological data. For example, Antarctic effects across the US Atlantic coast have been estimated at 1.3× the eustatic melt signal^16^. However, recent research suggests melting from Antarctica ceased or slowed substantially approximately 4000 years ago^17, 18^, and similar timing probably applies to Greenland. While eustatic effects could lead to over- or under-estimation of the vertical land motion with the geological approach, several studies suggests that these effects are small (<0.2 mm yr^−1^)^19, 20^. The good agreement between our GPS rates and the geological estimates over a substantial fraction of the east coast of North America confirms this^14^ (see also Fig. 1). Details concerning assumptions and uncertainties in the Holocene RSLR database can be found in refs 14 and 15. The current database consists of eighteen coastal sites in the US and southern Canada^14^.

**Figure 2:**
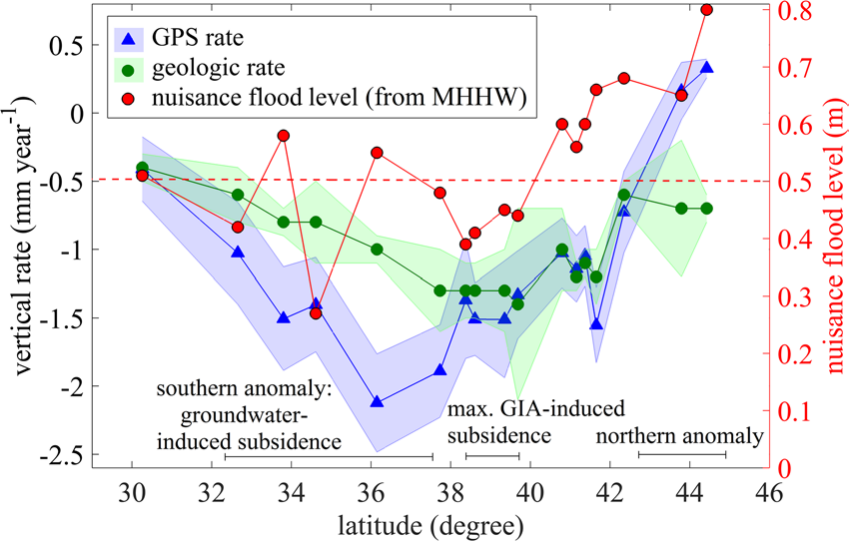
Comparison of nuisance flooding level (red circles), GPS vertical rate (blue triangles) and geological vertical rate (green circles) as a function of latitude along the US eastern seaboard. Shaded error bar is 1σ. The GPS rates and nuisance flooding level data are averaged for all stations and tide gauges in areas where geologic rate data are available (boxes in Fig. S1). Nuisance flood level is defined by NOAA as threshold flood elevation (a fixed height) above the 1983-2001 Mean Higher High Water (MHHW) tidal datum. Nuisance flooding is thus more frequent where the nuisance flood level is lower. The dashed red line marks an arbitrary 0.5 m nuisance flood level. Areas lower than this threshold (parts of our southern anomaly, and areas affected by GIA-induced subsidence) experience significant nuisance flooding, and are at greater risk of catastrophic flooding from storm surge. Possible effects of tidal range variations can be isolated by dividing the nuisance flood level by tidal range (Fig. S3).

Vertical land motions averaged over the two time scales are shown in Fig. 1. To facilitate comparison, we also average rates from all GPS stations within specific regions where Holocene RSL rise data are available (18 boxes in Fig. S1). As shown in Figs 1 and 2, the two data sets are in broad agreement. Both show the expected subsidence maxima centered near Chesapeake Bay (39° North), in agreement with GIA models (Fig. S2). These models describe the delayed response of the lithosphere to mass unloading associated with retreat of the Laurentide ice sheet starting about 20,000 years ago^21^. The subsidence maximum reflects the collapse of a peripheral bulge, which occurs just south of maximum glacier extent. The bulge is a typical mechanical response to loading and subsequent isostatic adjustment of a rigid or semi-rigid plate over a viscous or inviscid fluid substrate^22–25^. Its subsequent collapse leads to long-term subsidence^26^ and explains one of the areas experiencing a low threshold for nuisance flooding (Figs 2 and S3). The recent increase in flooding rate here reflects this susceptibility, combined with recent SLR.

There are two areas where the GPS (decadal) and geological (late Holocene) data show different rates. We call these areas the southern and northern anomalies, respectively. The southern anomaly is a broad region from 32.5°−37.5° North (Virginia, North and South Carolina), with substantial scatter in the GPS data. While some GPS sites agree with the geological data, other show high subsidence rates, at roughly twice the geological rate (Figs 1, 2 and S2). Parts of this area are also experiencing an increase in the frequency of nuisance flooding.

The northern anomaly is from 45°-43^°^ North (the states of Maine and New Hampshire) where the GPS data show uniformly slow uplift, in contrast to the geological data, which indicate slow subsidence. This area is not experiencing an increase in the frequency of nuisance flooding.

## Discussion and Conclusions

**Figure 3:**
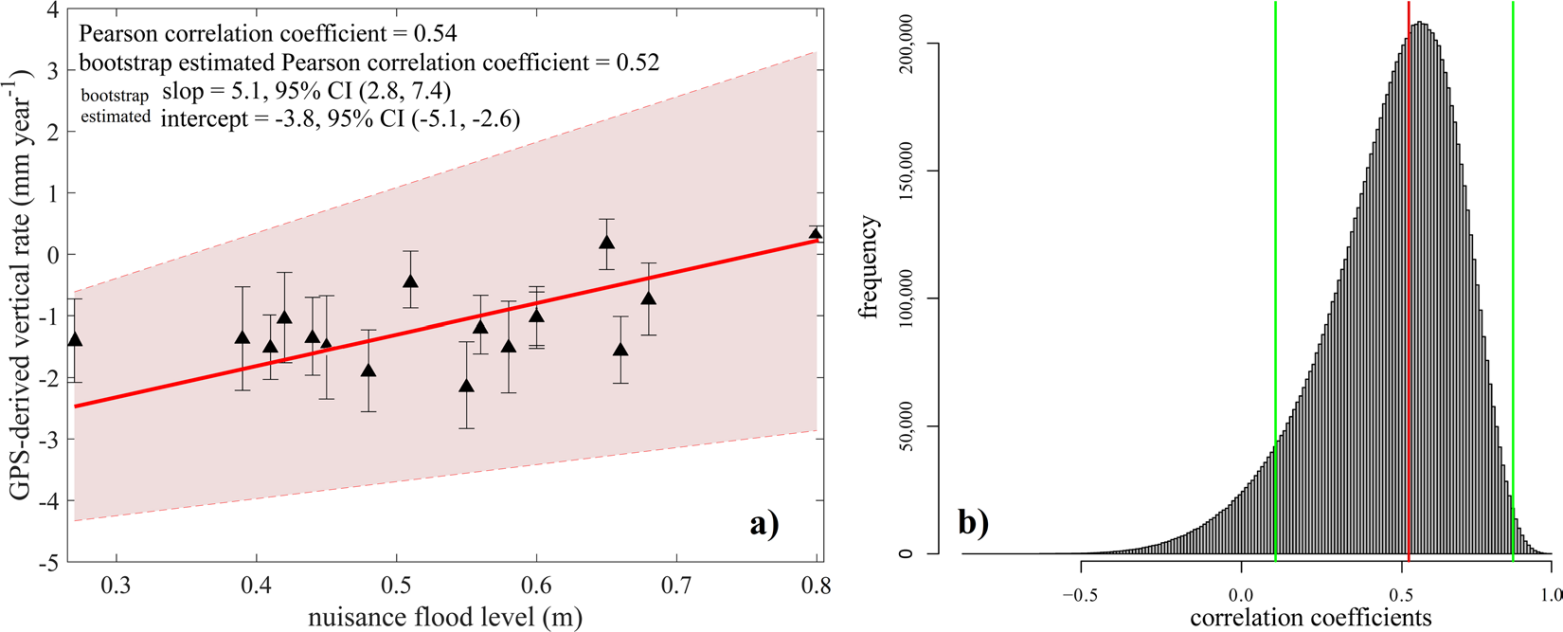
**(a)** Scatter plot of GPS-derived vertical rate compared to nuisance flooding level. The simple Pearson correlation coefficient and the bootstrap-estimated Pearson correlation coefficient (accounting for uncertainty in GPS rates) are 0.54 and 0.52, respectively. The red shaded area is 95% confidence interval for the regression line. Error bar are 1.96σ. The bootstrap estimated slop and 95% confidence interval is significantly different from zero. **(b)** Histogram showing bootstrap result for correlation coefficients. The red line represents the mean of the population (0.52) and the green lines bracket 95% of the estimates (0.10–0.84).

**Figure 4:**
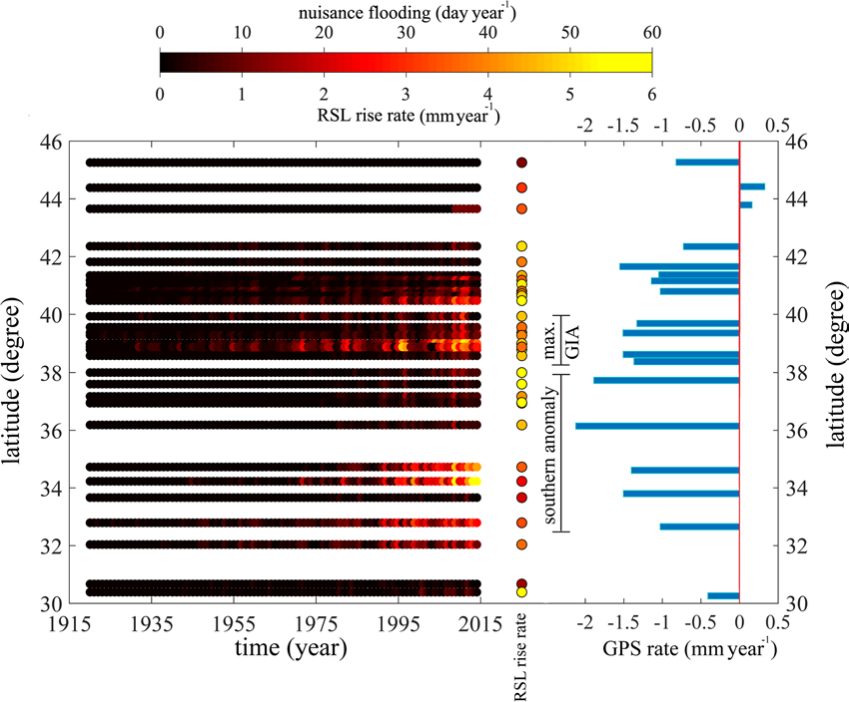
Nuisance flooding frequency versus time for various latitudes, rate of relative sea-level rise for 1990-present from tide gauges, and vertical rate from GPS. The flood frequency is defined as the number of days per year above a threshold flood level (nuisance flood level), for 34 tide gauges from St John’s in eastern Canada to Mayport, Florida. “max. GIA” refers to maximum subsidence due to the collapse of a peripheral bulge as observed by geologic data and GPS measurements. The southern anomaly refers to groundwater-induced subsidence as shown in Figs 2, S3 and S4.

Figure 2 compares GPS-derived rates of land motion to the nuisance flooding level. The latter is defined as a fixed height threshold elevation above the 1983–2001 MHHW (Mean Higher High Water) tidal datum at a given location, above which a rise in water level begins to impact lives, property, or commerce^27^. Thus, lower nuisance flood levels indicate a higher likelihood of flooding - the low elevation threshold is easily exceeded by even moderate flood events. The GPS and nuisance flood level data sets show moderate positive correlation (see also Fig. S3) with a Pearson correlation coefficient of 0.54 (*P*-value 0.02). We used a non-parametric bootstrap calculation to assess the influence of data uncertainty on the estimate of correlation coefficient, and to rigorously estimate the standard error and confidence interval of this estimate. We generate data subsets by random sampling from normal distributions with means equal to GPS rates and standard deviations equal to the rate uncertainty. We repeatedly estimate the Pearson correlation coefficient based on 10^7^ bootstrap resamples of the data points. The resulting distribution of correlation coefficient defines the 95% bootstrap confidence interval (Fig. 3b). The bootstrap estimated Pearson correlation coefficient (the mean of bootstrapped sampling distributions) is 0.52 with estimated bias of −0.05 and standard error of 0.21. These parameters are used to form the bias-corrected and accelerated (BCa) confidence interval^28^ to adjust for both bias (the skewness in the bootstrap sampling distribution) and non-normality of the sampling distribution. The BCa 95% confidence interval is 0.10 to 0.84. Even the lowest value of correlation coefficient at the 95% significance level is positive. Hence there is a likely connection between subsidence and nuisance flooding. The nuisance flooding database shows a local minimum in nuisance flooding level (i.e., lowest threshold elevation, highest likelihood of flooding) near the maximum subsidence rate associated with the collapse of the peripheral bulge. Recent increases in flood frequency are focused here (Figs 2, 4 and S3). This should not be surprising - much of the eastern seaboard is a low-slope coastal plain. Coastal settlements here were established close to sea level, hence even small increases in relative sea level will have a significant impact on flood frequency.

Although the peak subsidence rate from GIA (1.5 mm yr^−1^) is only half the current rate of global sea-level rise estimated from satellite altimetry^29^, it is the dominant factor in east coast nuisance flooding because it has been operating much longer, and thus has had the largest impact on *changes* in relative sea level. Many coastal towns in the region were established in the late 1600’s and early 1700’s. Those established near the GIA subsidence maximum have experienced approximately 0.45 meters of land subsidence from GIA (1.5 mm yr^−1^ × 300 years). Combined with 1.2 mm yr^−1^ of global sea-level rise for 90 years (1901–1990)^30^ (assumed insignificant prior to 1900) and the effects of recently accelerated global sea-level rise (ranging from 2.5 mm yr^−1^ to 3.4 mm yr^−1^) over the past two and a half decades, these urban centers have experienced a total of approximately 0.6 meters of RSLR since their establishment, with 75% of it due to GIA. This area may also be experiencing recent accelerated sea-level rise due to ocean dynamics and ice-mass loss from Greenland^31^. Increased frequency of nuisance flooding here (Fig. 4) is thus easy to understand. The combination of factors contributing to RSLR here also puts the region at greater risk from catastrophic flooding during storm surge events.

Nuisance flooding frequency shows a second maxima centered near 34° North, close to the southern anomaly defined by our GPS data (Figs 1 and 4). A recent study^31^ suggests ice-mass loss from Greenland, ocean dynamics and the inverted barometer effect explains accelerated sea-level rise along much of the East Coast of North America. However, this model produces a poor fit (see Fig. 4 of ref. 31) along our southern anomaly. Figures 1, 2 and 4 show that the GPS-measured subsidence rates, while variable, can be as high or higher here as they are in the “max GIA” anomaly. We suggest that the recent increased rate of RSLR here includes a contribution from subsidence of the land surface associated with recent groundwater loss, where pumping rates have exceeded the rate of natural recharge for a number of years^14^. The resulting loss of pore fluid pressure in the aquifer leads to compaction, loss of porosity, and surface subsidence. A database showing changes in groundwater level (Fig. S4) shows a large degree of spatial variability (aquifers tend to be locally managed) but also exhibits a broad minima (extreme drop in groundwater level) near 34° North, similar to the GPS-geological rate. This is encouraging for short-term (next few decades) mitigation, since groundwater management practices can be modified, reducing induced subsidence, and perhaps even promoting moderate uplift via groundwater recharge^32^.

In the area between 36° North and 38° North (south of Chesapeake Bay, Virginia) despite the higher rate of RSLR (tide gauge) and vertical land motion (GPS), the nuisance flooding frequency data show only a small increase (Fig. 4). Perhaps GPS-measured subsidence is sufficiently recent to have only a marginal effect on elevation. Our previous study^14^ shows that groundwater levels in most of the southern Chesapeake Bay region (south of Virginia) declined from the early 1970s until the late 2000s in response to excessive withdrawal but reversed from the late 2000s until 2015, indicating groundwater recharge (Fig. 4 of ref. 14). This indicates that groundwater-related subsidence can be a short-term phenomenon, and suggests that recent groundwater management efforts have been effective at reducing aquifer compaction and subsequent land subsidence in this area.

The northern anomaly is positive (GPS indicates slow uplift, differing by about 1 mm yr^−1^ from the geologic rate, which indicate slow subsidence) and does not appear to be related to recent changes in groundwater usage. The northern anomaly might reflect a modern peripheral bulge associated with recent loading by dams in northern Quebec, Canada^14^. The James Bay Project is a massive hydro-electric project involving construction of many dams on rivers draining into James Bay, Hudson Bay, and the Gulf of St Lawrence, constructed between the mid-1970’s and the late 1990s. Here we explore a quantitative test of this hypothesis.

**Figure 5:**
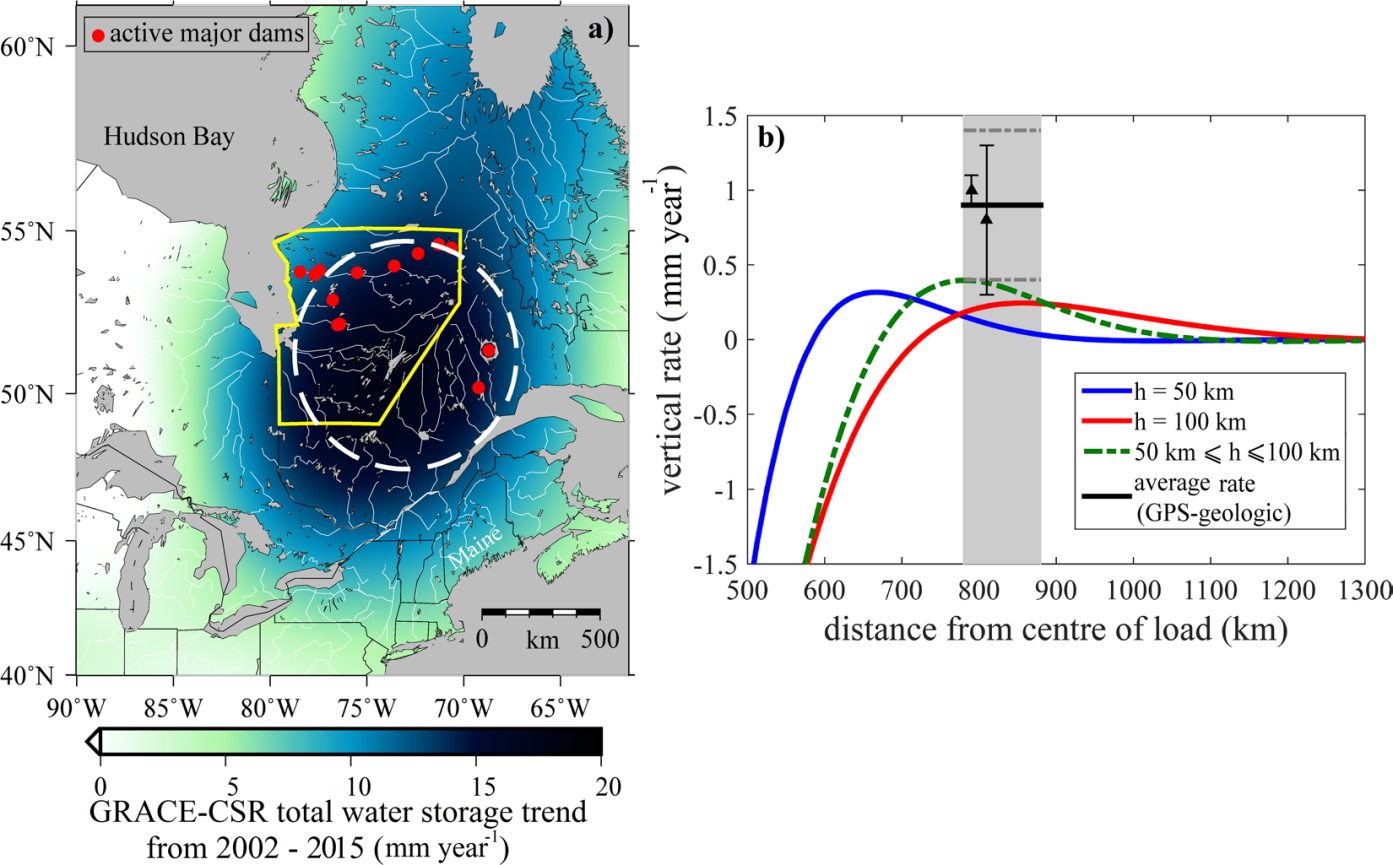
GRACE TWS and finite element model prediction. (**a**) Trend in total water storage (in equivalent water height) for eastern North America estimated from GRACE DDK2-filtered Stokes coefficients (corrected for GIA model^51^). The data represent an average for the period 2002–2015. The location of active major dams (red dots) and formal boundaries for the James Bay hydroelectric project (yellow line) are also shown. The white dashed circle with radius 400 km approximates the excess mass observed with GRACE. The mass anomaly is centered southeast of dams on rivers running northwest into James and Hudson Bay, and northwest of dams on rivers running southeast into the Gulf of St Lawrence. (**b**) Axisymmetric finite element model results for the peripheral bulge uplift rate in the vicinity of the northern anomaly induced by a load comparable to the total water storage estimated by GRACE for the James Bay Hydro-Electric Project in Quebec. The gray band shows distance of coast of Maine from the load centre. Triangles with error bar represent GIA-corrected vertical rate (GPS rate – geologic rate) for two regions where GPS and geologic rates are available^14^. Black line and dashed gray lines represent the average vertical rate (mean of two rates) and its uncertainty (1-σ error) along the northern anomaly. Dashed green line corresponds to a model where the elastic thickness (h) transitions from 100 km (elastic thickness for the Canadian Shield) for distances closer than 500 km, to 50 km (elastic thickness for the Appalachians) for distances farther than 780 km. Map is generated using GMT software version 5.1.0 (http://gmt.soest.hawaii.edu/)^66^.

Figure 5a shows the recent (2002–2015) change in Total Water Storage (TWS) in Quebec using data from the GRACE satellite gravity mission^33^. These data define the magnitude and approximate location of recent changes to water load, in this case concentrated southeast (upstream) from dams draining into James and Hudson Bay, and northwest of dams draining into the Gulf of St Lawrence. TWS is an estimate of total surface and near surface water stored on the continent, including groundwater, soil moisture, surface water, snow, ice, and biomass. Details of the GRACE processing and the TWS estimation are described in the Methods section. Although dam construction was completed prior to the start of the GRACE mission, reservoir filling continued for some time. To verify this, we also used satellite altimetry estimates for nine large lakes and reservoirs in the area (Figs 5a and S5). Time series of water-level changes here during part of the GRACE mission show significant positive trends, consistent with the GRACE observations (Figs S5, S6 and Table S2). While the main effect of this excess mass in the immediate area of the James Bay project is subsidence, distal areas experience uplift due to the peripheral bulge effect.

Figure 5b shows the modeled response of the lithosphere to this recent load, including the far field response along the coast of Maine. The model simulates the elastic (short-term) response of the lithosphere (an elastic plate with thicknesses between 50 and 100 km)^34, 35^ overlying a dense inviscid substratum. Details of the modeling procedure are described in the Methods section. The coast of Maine is ~ 780–880 km from the load maximum, close to the region of maximum uplift associated with the peripheral bulge predicted by the model, assuming elastic thickness transitioning from 100 km (elastic thickness for the Canadian Shield)^34^ to 50 km (elastic thickness for the Appalachians)^35^.

While the model correctly predicts the location of anomalous uplift, it underpredicts the amplitude by ~0.5 mm yr^−1^. This may be related to the uncertainty of GIA models used to correct GRACE TWS data^36^ (a range of 20% is often assumed for GIA models) and the greater uncertainty associated with geologic rates along the southern coast of Maine (±0.5 mm yr^−1^), but we cannot preclude the possibility of additional or alternate processes. Better understanding of these processes will be important for interpreting tide-gauge data in terms of future oceanographic changes. For example, rates of RSLR are lower in the northern anomaly than surrounding areas (Fig. 4).

Both nuisance flooding associated with periods of high tide, and catastrophic flooding associated with tropical storms and hurricanes, are increasing due to sea-level rise. While the latter cannot yet be predicted with any certainty, both the timing and location of nuisance flooding can in principle be predicted. In the short term, such predictions are useful in several ways. First, they can assist municipalities in mitigating the worst effects of flooding through improved infrastructure. Second, better management practices for extraction of groundwater from coastal aquifers can reduce nuisance flooding, by reducing or eliminating the coastal subsidence associated with over-extraction, temporarily reducing the rate of RSLR. On longer time scales however, many areas currently affected by nuisance flooding can expect to experience loss of coastal land unless significant infrastructure investments are made. Recognition of this fact can assist municipalities in making the necessary long-term plans and investments.

## Methods

### Analysis of GRACE data

Monthly total water storage (TWS) estimates were produced based on post-processing the Stokes coefficients (RL05) provided by the Center for Space Research (CSR), the Jet Propulsion Laboratory (JPL) and the GeoForschungsZentrum Potsdam (GFZ) for the period 2002–2015. We post-processed the non-isotropic filtered Stokes coefficients^37^ provided by ICGEM (http://icgem.gfz-potsdam.de/ICGEM/TimeSeries.html) as is typical, replacing zonal degree 2 Stokes coefficients (*C*_20_) with the more reliable solution from analysis of Satellite Laser Ranging (SLR) measurements^38^ and adding degree 1 Stokes coefficients (*C*_10_, *C*_11_ and *S*_11_) obtained from oceanic models^39^ available at the NASA-JPL Tellus website (ftp://podaac.jpl.nasa.gov/allData/tellus/L2/degree_1/). The spherical harmonic expansion was truncated at degree and order 60. These coefficients are non-isotropic filtered Stokes coefficients (DDK2) where the isotropic part resembles a Gaussian filter with a half width of 340 km^40^, corresponding approximately to the isotropic filter used in producing the NASA-JPL Tellus gridded GRACE TWS data.

We also used post-processed gridded TWS data provided by the NASA-JPL Tellus website (https://grace.jpl.nasa.gov/data/get-data/monthly-mass-grids-land/) to compare the magnitudes of TWS trends with those estimated using the DDK2 filter. Recent studies have used the Tellus GRACE TWS products in hydrology^41–47^. The Stokes coefficients are smoothed using de-striping filter^48^ and a 300 km wide isotropic Gaussian filter^49^. The post-processing algorithms are detailed in the literature^48, 50^. The Tellus gridded GRACE TWS data are corrected for GIA effects based on a model^51^, which uses ICE-5G^52^ for ice loading history and a compressible Earth model. We smoothed the GIA model using the DDK2 filter and corrected the DDK2-derived GRACE TWS trend.

The trend estimates based on DDK2-filtered Stokes coefficients and the Tellus gridded GRACE TWS data from three processing centers (CSR, GFZ and JPL) are compared in eastern Canada (Fig. S7 and Table S1). Although the processing centers use different algorithms to produce Stokes coefficients, the TWS trends from all three processing centers are very similar over eastern Canada. The trends based on DDK2 Stokes coefficients show a similarity in shape but are slightly larger (3–4 mm yr^−1^) relative to trends estimated from the Tellus gridded GRACE TWS data.

The loading signal from a single water reservoir typically has a wavelength much shorter than the GRACE spatial resolution. Loads from dams are often modeled as point sources, especially in high relief terrain where the reservoir is spatially compact. In this case the transfer function from water storage to radial displacement has little power^53^. However, the James Bay project consists of multiple dams and reservoirs on a broad Precambrian peneplain, covering thousands of square kilometers. The total rate of mass increase from just the nine reservoirs shown in Fig. S5 where altimetry is available is about 1.7 km^3^ yr^−1^, explaining about 30–40% of TWS changes trend observed by GRACE. The excess mass observed in Quebec with GRACE has a radius of ~ 500 km (the dark blue region in Fig. 5a). This includes real mass increases due to increased groundwater and soil moisture in areas surrounding the reservoirs, but also includes some leakage effects due to filtering and truncating Stokes coefficients. We therefore model the excess mass as a uniform flat disc load (simulated as a uniform pressure) with a smaller radius, 400 km (white circle in Figs. 5a and S7) to account for the leakage effect. Given the low pass filtering behavior of an elastic lithosphere, the details of the load geometry are not important, and this approach is probably adequate.

The TWS observed with GRACE includes the sum of soil moisture, groundwater and surface water (including rivers, dams, lakes and snow). Surface water is probably the main contributor, and its relative contribution be estimated by looking at hydrollgical models for the other components. Figure S8 shows the sum of soil moisture and groundwater trends estimated from the WaterGap Global Hydrological Model (WGHM, version 2.2b)^54, 55^. These components of water storage show a small trend, ranging from −1.7 to 1.7 mm yr^−1^ over the James Bay hydroelectric project and its adjacent areas. Thus, the excess mass observed with GRACE is primarily stored as surface water in rivers, dams and lakes.

### Modeling the far field effects of water loading from the James Bay Project

Construction of the James Bay project began in the mid-1970’s and was largely complete by the late-1990’s. The project had two main phases, Phase 1 from the mid-1970s to the mid-1980’s, and Phase 2 from the late 1980’s to 1996. Filling of the reservoir with surface water continued into the early or mid-2000’s, but increases in groundwater would likely continue for a much longer period given the low permeability of Precambrian Shield rocks. GRACE began to collect data in 2002, and hence covers the later phases of reservoir filling and groundwater increase. This likely explains slightly negative acceleration in TWS data observed in Quebec^56^. Our modeling uses the observed rate of mass change as measured by GRACE, and hence predicts current rates of surface deformation, assuming elastic deformation. The load is centered in the middle of the excess mass observed by GRACE (blue region in Fig. 5a) southeast of James Bay – Hudson Bay dams, the region of maximum flooding, i.e., upstream from these dams.

We present a numerical solution for the deformation of a thin elastic plate of uniform or tapering thickness overlying an inviscid dense substratum (a fluid with zero viscosity) with a density of *ρ*_*s*_ = 3300 kg/m^3^. The condition of static force equilibrium (i.e., between the surface load *P*, the elastic response of the plate and the response of the fluid substratum on the base of the plate) for a thin plate on a fluid substrate results in a fourth-order differential equation^57, 58^:

1 $${\rho }_{s}\,gw(r)+D{\rm{\Delta }}{\rm{\Delta }}w(x,y)=P$$

where Δ is the Laplacian operator, *w*(*r*) is the vertical deflection of the plate at a distance *r* from centre of load and *D* is a plate flexural rigidity which depends on the elastic rigidity *μ*, plate thickness *h* and the Poisson’s ratio *ν*:

2 $$D=\mu {h}^{3}/6(1-\nu )$$

The load *P* is approximated with a disc of height *d*:

3 $$P={\rho }_{w}\,gd$$

where *ρ*_*w*_ = 1000 *kg m* ^−3^ is the water density and *g* = 9.8 *m s* ^−2^ is the gravitational acceleration. Our solution is based on numerical evaluation of equation (1) using the Finite Element Modeling (FEM) code GTecton^59^ in the axisymmetric version^60^. The response of the fluid substratum at the base of the plate (the term *ρ*_*s*_ *gw*) is assumed to be Winkler type in equation (1), thus the isostatic adjustment is estimated through the use of Winkler restoring forces^61^. The shear modulus and Poisson’s ratio are taken as *μ* = 30 GPa and *ν* = 0.3 respectively, and we let the plate thickness vary. We tested thicknesses between 50 and 120 km^34, 35^ compatible with the elastic thickness estimated for the Appalachians (the smaller number) or the Canadian Shield (the larger number), as well as a transitional model, tapering between the two thicknesses. As mention earlier, we model the excess mass observed in Quebec as a disc-shape constant load with radius 400 km and a mean thickness rate $\dot{d}$ = 18 mm yr^−1^.

Although our model of a thin elastic lithosphere over an inviscid mantle and its response to GRACE-derived loading can explain the location of uplift along the Maine shoreline, there is an inherent inconsistency in our approach. In calculating TWS from GRACE Stokes coefficients (conversion from potential anomaly to surface mass anomaly), a series of dimensionless load Love’s numbers (LLNs) are used to account for the effects of solid earth deformation^49^. The LLNs represents the static deformation of a spherically-symmetric non-rotating elastic isotropic Earth model^62^ such as the Preliminary Reference Earth Model (PREM)^63^. The PREM model assumes an elastic crust and mantle with radial variations of elastic parameters and density based on a linear Maxwell solid rheology. Thus, there is an inconsistency in the rheological assumptions used in the calculation of TWS from GRACE Stokes coefficients compared to the rheological assumptions used in our modeled response to surface loading, which includes a thin lithosphere over an inviscid mantle. While our approach provides a first-order approximation of lithospheric peripheral bulge effects associated with recent loading by dams in northern Quebec, Canada, a more detailed model, and a more consistent approach between GRACE-derived data and deformation model, is warranted.

### Data availability

Late Holocene RSL data and GPS data on the rates of vertical land motion are publicly available^14, 15^. Data describing nuisance flooding level and nuisance flooding frequency are available from NOAA National Weather Service (http://water.weather.gov/ahps/) and other studies^2^. Changes in local groundwater levels are available as well data from the U.S. Geological Survey groundwater information page (https://waterdata.usgs.gov/nwis), the North Carolina Division of Water Source (http://www.ncwater.org/?page = 343), and the South Carolina Department of Natural Resources (http://www.dnr.sc.gov/water/hydro/groundwater/groundwater.html). Tide- gauge data were obtained from NOAA (http://tidesandcurrents.noaa.gov/).

## Electronic supplementary material

SUPPLEMENTARY INFO
