# Supplementary material for: Nuisance Flooding and Relative Sea-Level Rise: the Importance of Present-Day Land Motion
Source: Sci Rep. 2017 Sep 11;7:11197. doi: 10.1038/s41598-017-11544-y (PMC5593944; doi:10.1038/s41598-017-11544-y)
Supplement: Supplementary file 1 — SUPPLEMENTARY INFO [file 41598_2017_11544_MOESM1_ESM.pdf]

SUPPLEMENTARY INFORMATION

Nuisance Flooding and Relative Sea-Level Rise: The Importance of Present-Day Land Motion

*Makan A. Karegar[1,2], Timothy H. Dixon[1], Rocco Malservisi[1], Jürgen Kusche[2], [3]Simon E. Engelhart

[1] School of Geosciences, University of South Florida, Tampa, Florida, USA,

[2] Institute of Geodesy and Geoinformation, University of Bonn, Bonn, Germany.

[3] Department of Geosciences, University of Rhode Island, Kingston, Rhode Island, USA

* corresponding author

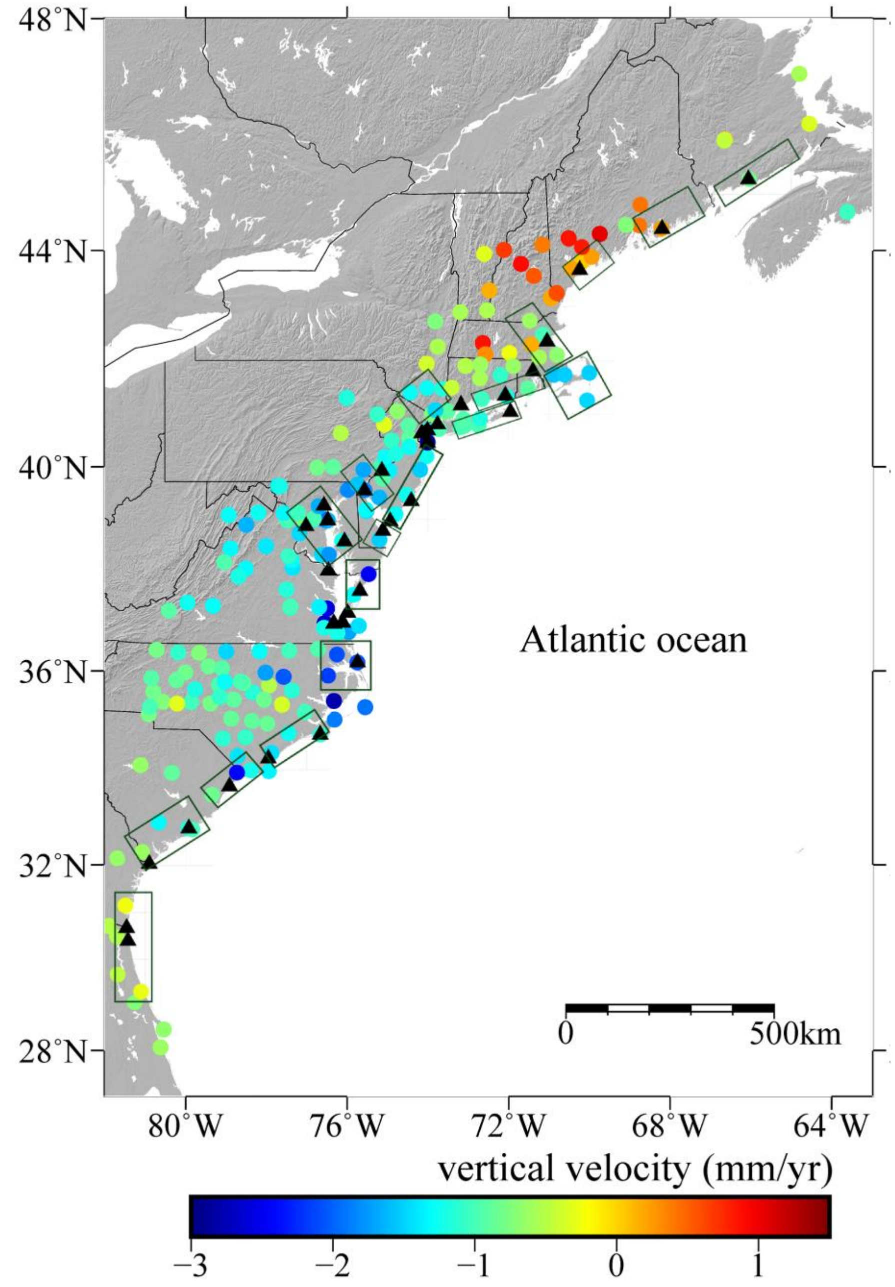

Figure S1. Map showing the location of GPS sites and 18 regions (boxes) for which the geologic rate is known. Circle color indicates decadal average vertical land motion in IGb08 reference frame. Black triangles are locations of tide gauges for which nuisance flooding level data and nuisance flooding frequency are available. The GPS rates and nuisance flooding level data shown in Figure 2 and Figure S2-S5 are average values for all stations in the boxes. Map is

generated using GMT software version 5.1.0 (http://gmt.soest.hawaii.edu/) (*Wessel* et al. 2013). Modified from *Karegar* et al. (2016).

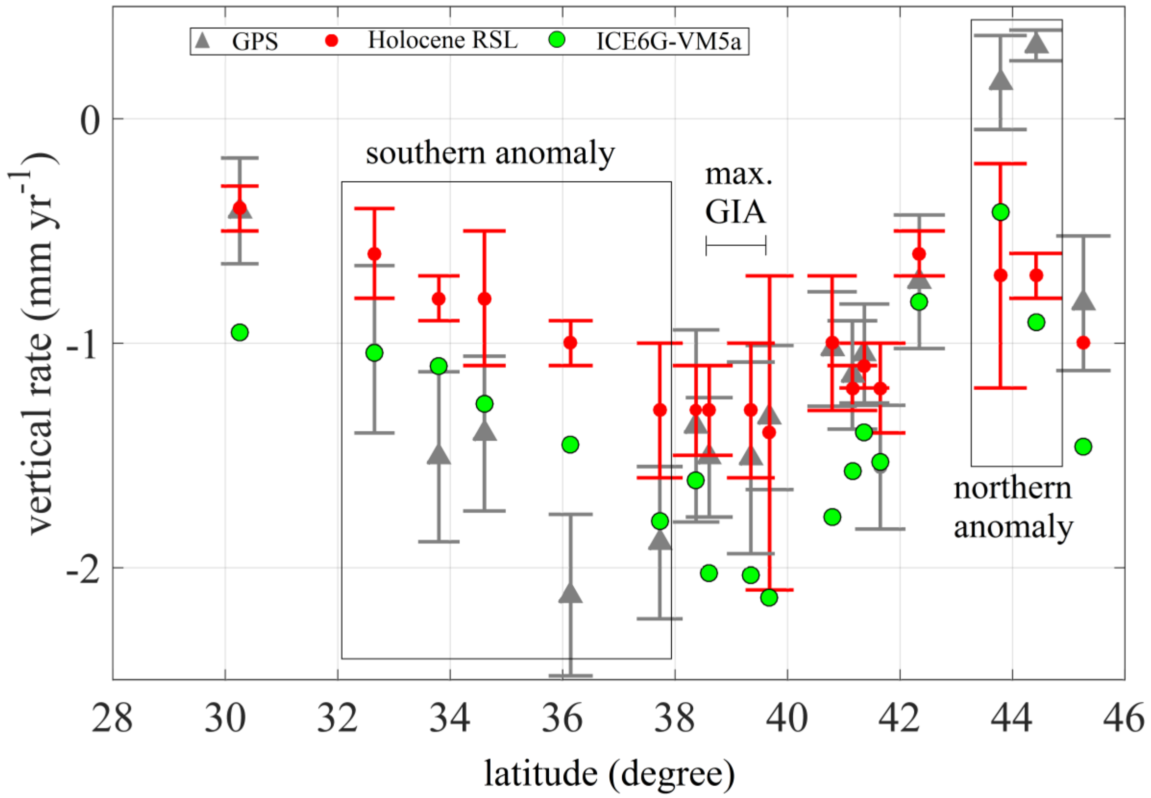

Figure S2. Comparison of spatially averaged GPS (gray triangles), geologic data (red circles), and GIA model ICE6G-VM5a (green circles) for eighteen coastal sites in the US and southern Canada (Figure S1). Error bars are 1σ.

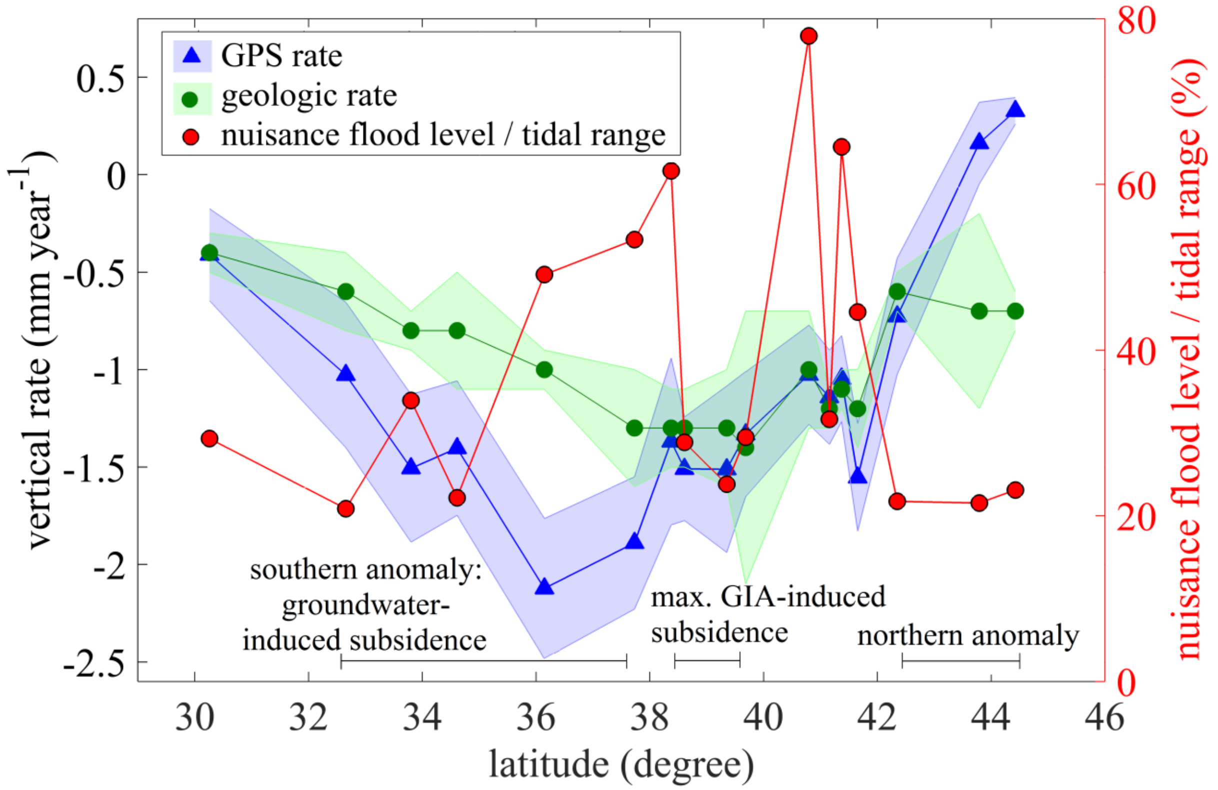

Figure S3. Comparison of nuisance flood level (red circles) as standardized by tidal range (MHHW- MLLW), GPS rate (blue triangles) and geologic rate (green circles) as a function of latitude along the US eastern seaboard. Figure S3 is an alternative plot to Figure 2. Here the possible effects of tidal range variations are isolated by dividing the nuisance flood level (measured from MHHW) and tidal range. As in Figure 2, the same relationships are seen between nuisance flood level and GPS rate. Note that the GPS rates and standardized nuisance flooding level data are averaged for all stations and tide gauges in the boxes shown in Figure S1 where geologic data are available.

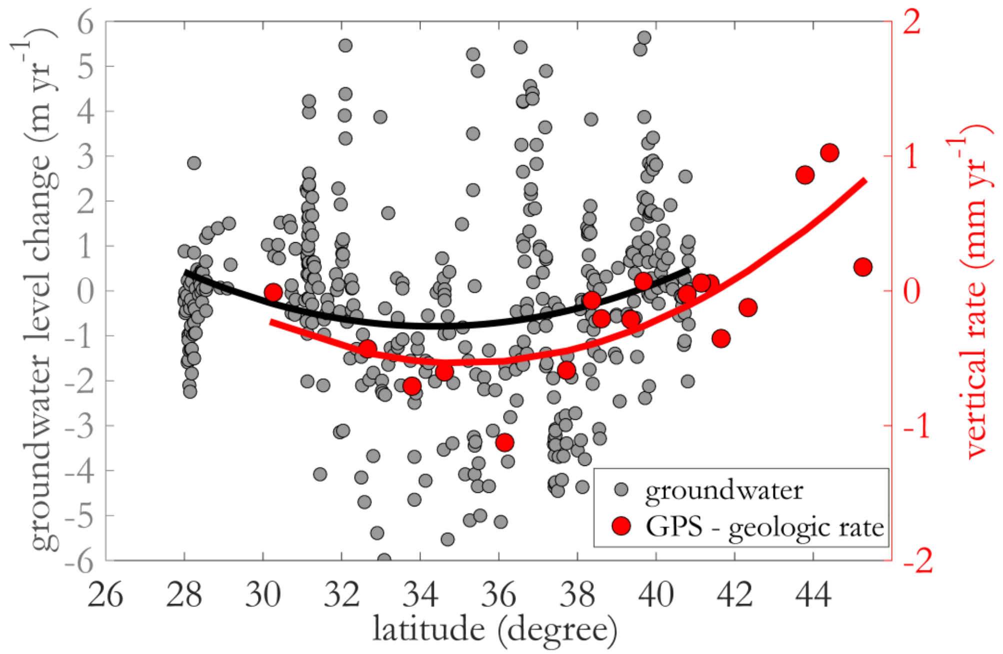

Figure S4. Comparison of GIA-corrected GPS-derived vertical rate (red dots) and average trend in groundwater-level changes (gray dots). The black and red solid curves are quadratic polynomials fit to the groundwater and vertical rate data, respectively.

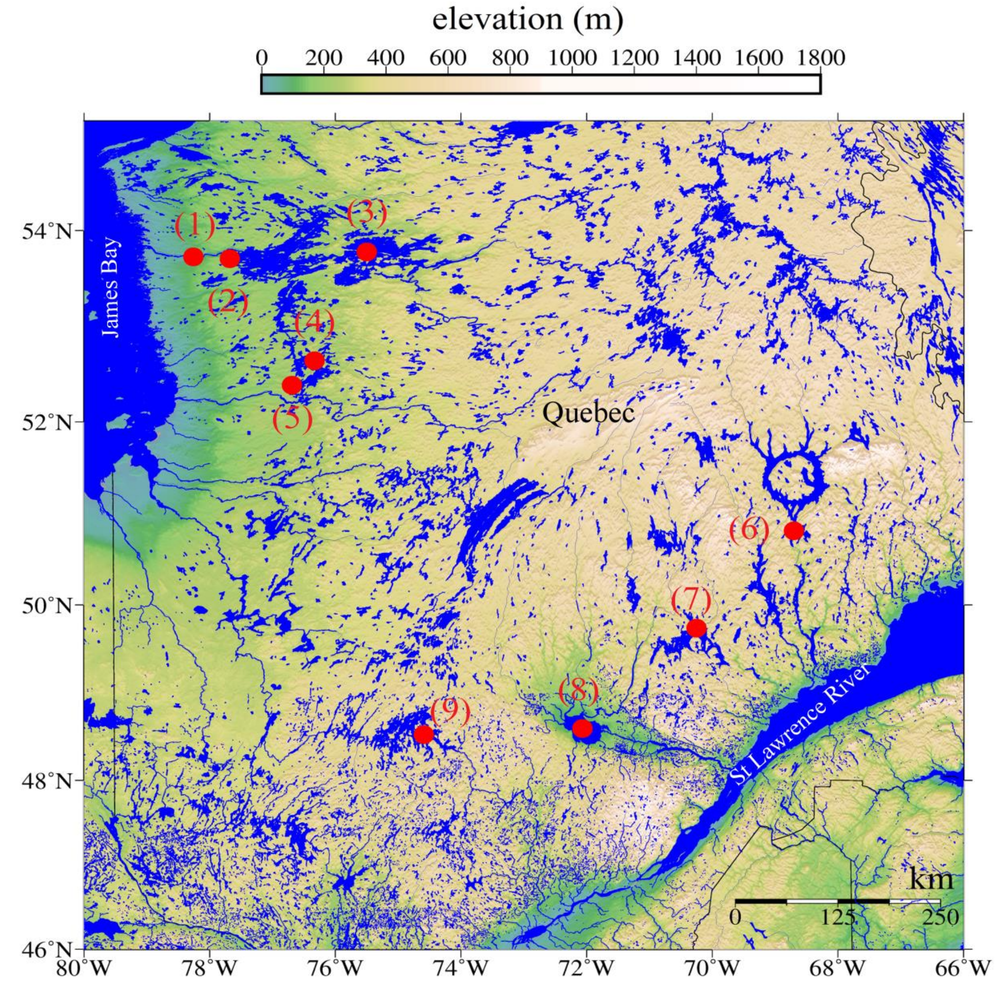

Figure S5. Map of Lake-Dam system in Quebec, Canada. Lakes with water-level data (virtual gauges) are numbered. For time series see Figure S6. Lake area and annual rate of water-level change are listed in Table S2. Map is generated using GMT software version 5.1.0 (http://gmt.soest.hawaii.edu/) (*Wessel* et al. 2013).

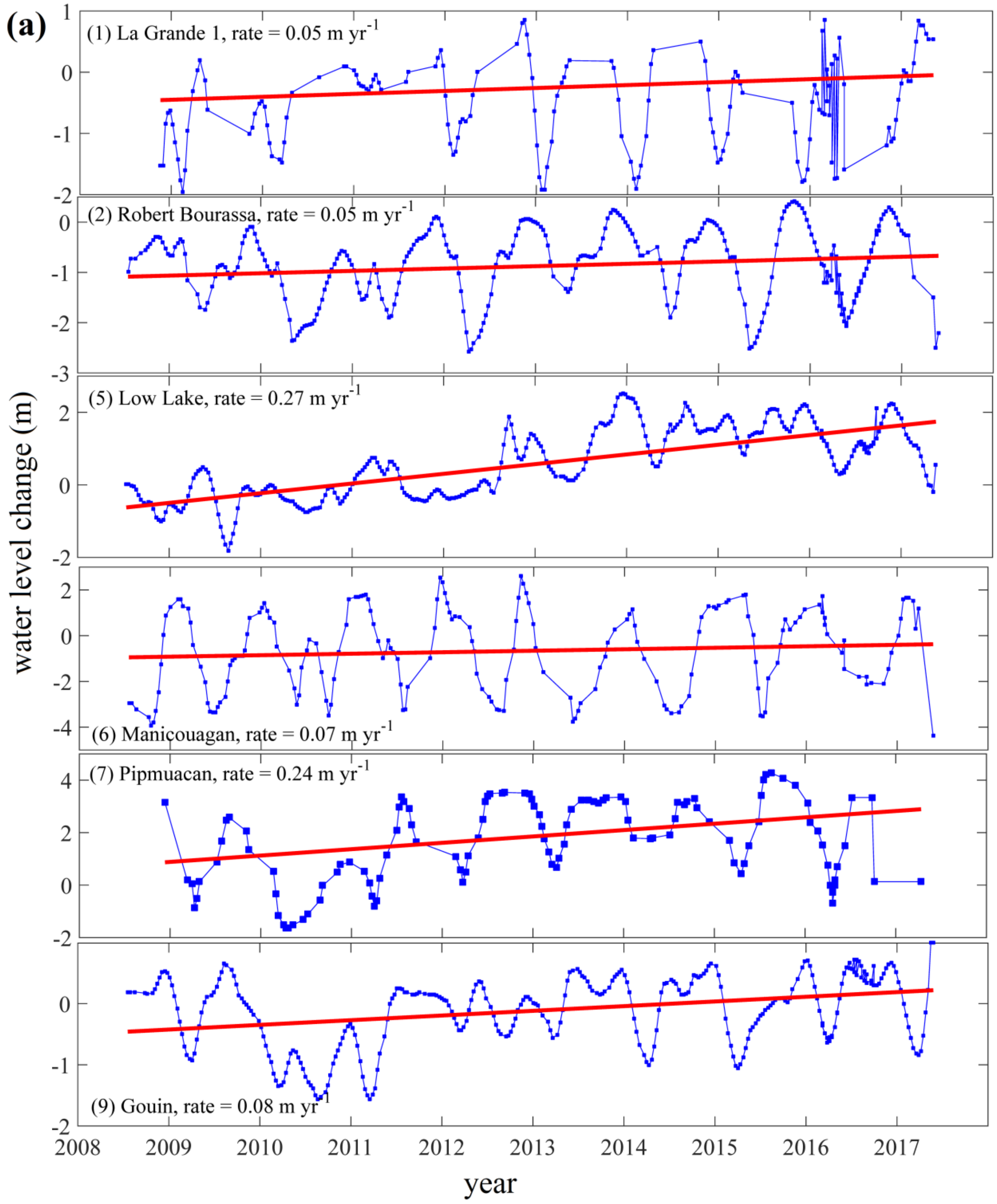
(a)
(1) La Grande 1, rate = 0.05 m yr-1
(2) Robert Bourassa, rate = 0.05 m yr-1
(5) Low Lake, rate = 0.27 m yr-1
(6) Manicouagan, rate = 0.07 m yr-1
(7) Pipmuacan, rate = 0.24 m yr-1
(9) Gouin, rate = 0.08 m yr-1
water level change (m)
year
2008
2009
2010
2011
2012
2013
2014
2015
2016
2017

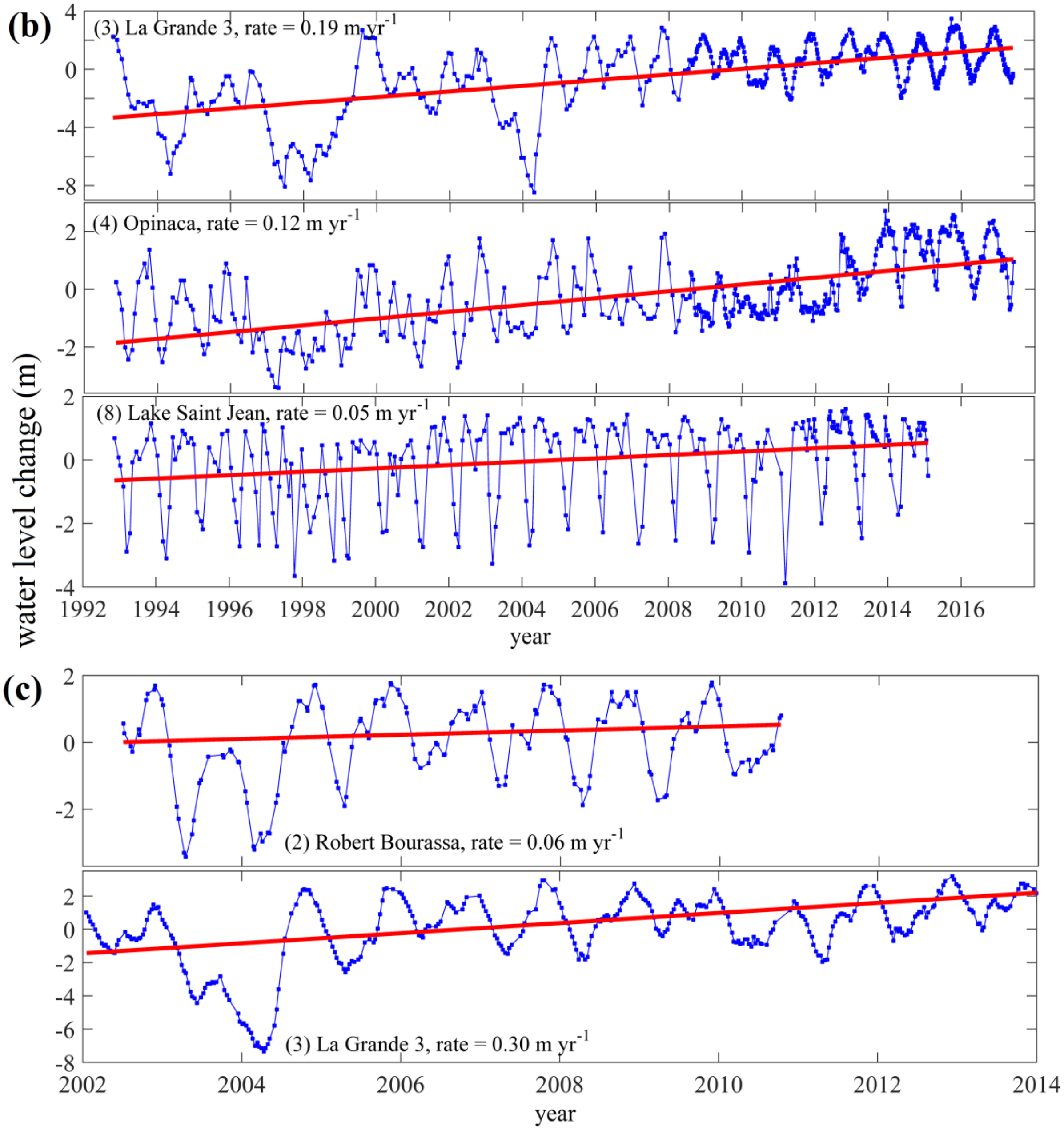

Figure S6. Time series of water-level change from satellite altimetry measurements produced by different processing centers. Lake level products are courtesy of **(a)** USDA/NASA G-REALM. **(b)** database for Hydrological Time Series of Inland Waters (DAHITI) **(c)** HYDROWEB database from Legos and THEIA platform. A least squares model fit was used to

define the rate. Model parameters include an initial offset, a constant rate and fixed amplitude annual variation.

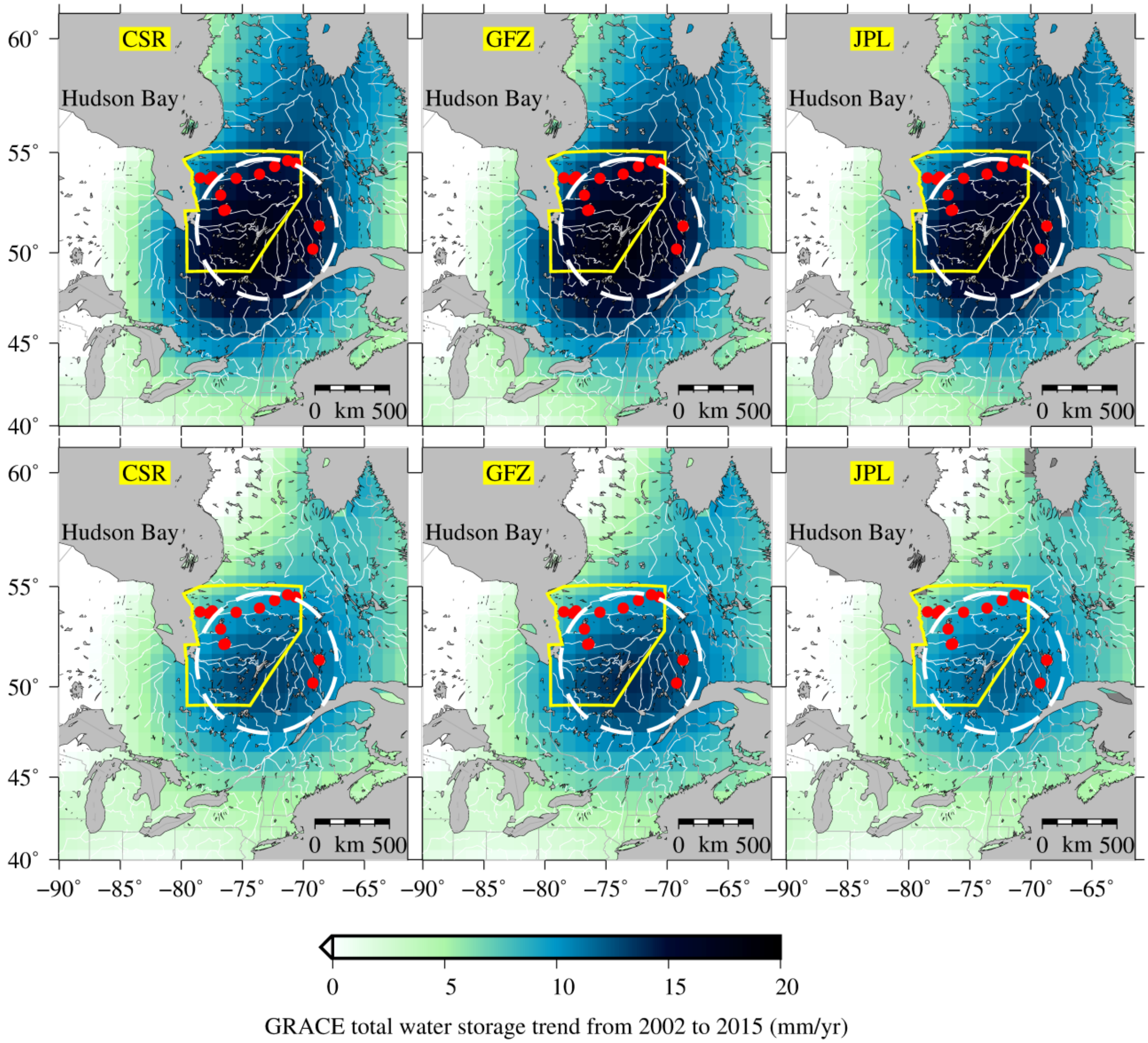

Figure S7. Comparison of trend in total water storage (in equivalent water height) from three GRACE solutions: The University of Texas Center for Space Research (CSR), Geoforschungszentrum (GFZ), and NASA Jet Propulsion Lab (JPL) (**Top panel**) GRACE

Stokes coefficients were smoothed by applying the non-isotropic filter DDK2 (*Kusche*, 2007). (**Bottom panel**) Post-processed gridded monthly TWS data provided by NASA JPL Tellus were used. Trend is corrected for the GIA model of *Geruo* et al. (2013). The location of active major dams (red dots) and formal boundaries for the James Bay hydroelectric project (yellow line) are also shown. The white dashed circle with radius 400 km approximates the excess mass observed in Quebec with GRACE. Map is generated using GMT software version 5.1.0 (http://gmt.soest.hawaii.edu/) (*Wessel* et al. 2013).

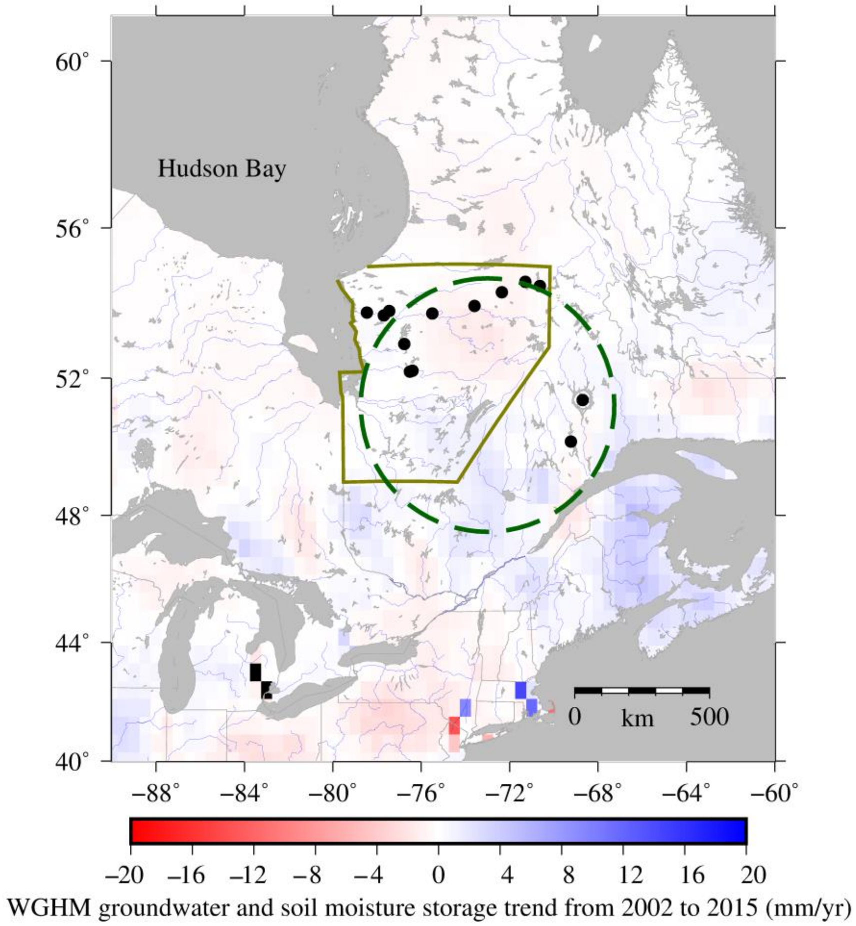

Figure S8. Sum of groundwater and soil moisture storage trend (in equivalent water height) from WaterGap Global Hydrological Model (WGHM, version 2.2b) (*Döll* et al. 2014; *Müller Schmied*

et al. 2014). Map is generated using GMT software version 5.1.0 (http://gmt.soest.hawaii.edu/) (*Wessel* et al. 2013).

Table S1. Statistics of GRACE TWS trends (in equivalent water height) based on three GRACE solutions (CSR, GFZ, and JPL) and two post-processing products including Tellus gridded GRACE TWS data (isotropic filter: Gaussian + de-stripping filters) and non-isotropic filter (DDK2 filter). Unit: mm yr^{-1}.

**Solution (G+De[1]/DDK2)**	**Min.**	**Max.**	**Mean**	**RMS**
**CSR**	-6.7/-9.9	13.1/17.9	4.3/6.1	6.0/8.5
**GFZ**	-6.6/-11.0	13.9/18.5	4.7/6.3	6.4/8.8
**JPL**	-6.2/-10.8	12.0/17.5	4.7/6.1	6.3/8.5

[1] G: Gaussian filter, De: de-stripping filter

Table S2. Characteristics of lakes and annual rate of water-level change from different satellite altimetry missions and processing centers. The non-parametric Mann-Kendall trend test was applied to the water-level change time series. The *P*-value of the two-tailed test at the significance level of 0.05 is listed. The trends are considered statistically significant when the p value falls below a critical value ($P < 0.05$). It is sufficient to conclude that there is a positive trend in the water-level variations for different periods. The total rate of change of water volume from the nine reservoirs listed in Table S2 is about 1.7 km^3 yr^{-1} which is ~ 30% - 40% of TWS changes observed by GRACE. Columns 6 and 7 are rate of water-level change and rate of volume change, respectively.

No.	Name	Area (km^2)	Altimetry data period	Satellite mission	Rate (m yr^{-1})	Rate (km^3 yr^{-1})	*P*-value
1	La Grande[1]	78	2009 - 2017	Jason 2 & 3	0.05	0.004	0.01
2	Robert Bourassa[1]	2,900	2008 - 2017	Jason 2 & 3	0.05	0.15	0.03
2	Robert Bourassa[3]	2,900	2002 - 2011	Envisat	0.06	0.17	0.06
3	La Grande[2]	2,500	1992 - 2017	T/P, Envisat GFO, Jason 2 & 3	0.19	0.47	0
3	La Grande[3]	2,500	2002 - 2014	Jason 1 & 2	0.30	0.74	0
4	Opinaca[2]	1,100	1992 - 2017	T/P, Envisat, Jason 2 & 3	0.13	0.12	0
5	Low Lake[1]	200	2008 - 2017	Jason 2 & 3	0.27	0.06	0
6	Manicouagan[1]	1,950	2008 - 2017	Jason 2 & 3	0.07	0.14	0.02
7	Pipmuacan[1]	980	2008 - 2017	Jason 2 & 3	0.24	0.24	1.7E-04
8	Lake Saint Jean[2]	1,053	1992 - 2014	T/P, Envisat GFO, Jason 2	0.05	0.05	9.3E-10
9	Gouin[1]	1,600	2008 - 2017	Jason 2 & 3	0.08	0.13	2.3E-08

Lake level products are from 1. USDA/NASA G-REALM program (https://www.pecad.fas.usda.gov/cropexplorer/global_reservoir/); 2. database for Hydrological Time Series of Inland Waters (DAHITI) developed by the Deutsches Geodätisches Forschungsinstitut der Technischen Universität München (DGFI-TUM) (http://dahiti.dgfi.tum.de/en/map/) (*Schwatke* et al. 2015); and 3. HYDROWEB database from Legos and THEIA platform (http://hydroweb.theia-land.fr/) (*Crétaux* et al. 2011).

Supplemental References

Crétaux, J. F., Jelinski, W., Calmant, S., Kouraev, A., Vuglinski, V., Bergé-Nguyen, M., ... & Maisongrande, P. SOLS: A lake database to monitor in the Near Real Time water level and storage variations from remote sensing data. *Advances in space research*, ***47***, 1497-1507 (2011).

Döll, P., Fritsche, M., Eicker, A., & Schmied, H. M. Seasonal water storage variations as impacted by water abstractions: comparing the output of a global hydrological model with GRACE and GPS observations. *Surveys in Geophysics,* **35,** 1311-1331 (2014).

Geruo, A., Wahr, J., & Zhong, S. Computations of the viscoelastic response of a 3-D compressible Earth to surface loading: an application to Glacial Isostatic Adjustment in Antarctica and Canada. *Geophysical Journal International*, **192,** 557-572 (2013).

Karegar, M. A., Dixon, T. H., & Engelhart, S. E. Subsidence along the Atlantic Coast of North America: Insights from GPS and late Holocene relative sea level data. *Geophysical Research Letters*, **43,** 3126-3133 (2016).

Kusche, J. Approximate decorrelation and non-isotropic smoothing of time-variable GRACE-

type gravity field models. *Journal of Geodesy*, **81,** 733-749 (2007).

Müller Schmied, H., Eisner, S., Franz, D., Wattenbach, M., Portmann, F. T., Flörke, M., & Döll, P. Sensitivity of simulated global-scale freshwater fluxes and storages to input data, hydrological model structure, human water use and calibration. *Hydrology and Earth System Sciences*, **18,** 3511-3538 (2014).

Schwatke, C., Dettmering, D., Bosch, W., & Seitz, F. DAHITI–an innovative approach for estimating water level time series over inland waters using multi-mission satellite altimetry. *Hydrology and Earth System Sciences*, ***19***, 4345-4364 (2015).

Wessel, P., Smith W.H.F., Scharroo R., Luis J.F. & Wobbe F. Generic Mapping Tools: Improved version released. *EOS Trans. AGU*, ***94***, 409- 410 (2013).
